# The effect of placebo on endurance capacity in normal weight children – a randomized trial

**DOI:** 10.1186/s12887-019-1394-x

**Published:** 2019-01-10

**Authors:** Shira Fanti-Oren, Daphna Birenbaum-Carmeli, Alon Eliakim, Michal Pantanowitz, Dan Nemet

**Affiliations:** 1 Cheryl Spencer Department of Nursing, University of Haifa, Haifa, Israel; 2 Pediatric Department, Child Health Sport Center, Meir Medical Center, Sackler School of Medicine, Tel Aviv University, Tel Aviv, Israel

**Keywords:** Sham effect, Information, Physical activity, Fitness

## Abstract

**Background:**

The aim of the study was to examine the influence of the placebo effect on the endurance capacity results in normal weight children.

**Methods:**

Twenty-four pre-pubertal normal-weight children aged 6–13 years participated in the study. Subjects underwent anthropometric measurements (weight, height, BMI percentile, and fat percentage), a progressive treadmill exercise test to evaluate endurance capacity, and filled habitual activity questionnaire. The participants were examined twice, in a random order, with each child being compared to him/herself. Different types of information were provided regarding a water drink consumed prior to testing- standard information (water) vs. deliberate positive information (presumed energy drink, placebo**).**

**Results:**

Following the placebo drink, children demonstrated significantly higher peak pulse (177.9 ± 13.6 vs. 189.8 ± 12.2 bpm), higher stage achieved and longer time of exercise to exhaustion (700.1 ± 155.2 vs. 893.3 ± 150.1 s). Although the exercise duration was longer, stage and heart rate achieved were higher, the reported average, and peak rate of perceived exertion (RPE) were significantly lower for the placebo (18.3 ± 1.4 vs 16.2 ± 1.5). Although the effort was higher while drinking placebo (longer run, higher exercise phase, higher heart rate), recovery time was significantly shorter. The reported differences were not associated with order of tests, age, gender or child activity level.

**Conclusion:**

Our results demonstrate a significant information placebo effect on children’s endurance capacity test results. This highlights the possible role of positive information (placebo) in trying to encourage physical activity in children. Whether this effect could be applied to longer-term interventions has yet to be tested.

**Trial registration:**

ClinicalTrial.gov identifier: NCT03165604, Registered May 24, 2017.

## Background

Placebo is a sham treatment—an inert substance or procedure that simulates a substance or procedure with an active effect material component. Its power rests in the patient’s perception instead of a scientifically known effective substance or procedure [1]. A placebo can be a pill, an injection, a drink, an operation, an exercise, a situation or even a piece of information. The term *placebo effect* describes the positive psychological and physiological changes to an individual when they believe they are receiving a treatment with a scientifically active component, and the belief that the treatment indeed causes an actual effect to the patient, whether in addition to the inherent effect of a truly potent treatment and whether exclusively, in the case of a placebo [2].

Placebos have been used for a long time as a methodological tool in clinical trials in order to isolate the physiological effect of the examined medication from its potential psychological impact. A group receiving the medication is compared to a control group receiving a placebo in order to isolate the placebo effect and focus solely on the biochemical effect associated with the medication [2].

Over the past few decades, there has been an increasing scientific interest in the placebo effect and its own potential as an add-on to medical treatment [3]. The placebo effect has rarely been tested in children, apparently due to the ethical difficulties raised by such research. However, the few existing reviews and meta-analyses usually conclude that the placebo response rates in trials are higher in children and adolescents than in adults despite the drug responses being equal [4]. The majority of clinical trials dealing with the placebo response in children have focused on attention deficit disorders and a handful of chronic illnesses such as migraines, depression, and epilepsy [4]. An important area in the field of the placebo effect in children that has yet to be studied to its fullest potential is physical activity.

Physical activity during childhood influences the growth and development of muscle tissue, fat, and bone; it constitutes an important physiological stimulus for the secretion of growth hormones in children, and it allows for proper child growth and development [5]. In recent years a persistent decline in physical activity levels is observed in children, with most children today not meeting the WHO guidelines for physical activity [6, 7].

The present study (identifier: NCT03165604) examines the placebo effect on endurance capacity assessed by a progressive running treadmill test in children. To the best of our knowledge, this is the first study to date that has undertaken a systematic measurement of the placebo effect in children and in the context of physical activity. Like the previously depicted studies, this research has employed a placebo in the form of information and examined its influence on the results of aerobic stress tests and on the subjects’ perception of the effort. We hypothesized that providing the children with positive guiding information will lead to immediately improved endurance capacity test results and to an immediate decrease in the children’s rate of perceived exertion (RPE).

## Methods

### Participants and recruitment

Twenty-four (12 girls and 12 boys) normal weight children participated in the study following informed consent signed by their parents/guardians. Participants’ characteristics are presented in Table 1. Twenty-five participants were initially recruited between July 2017 and May 2018, one participant had an unrelated to the study fracture and was unable to complete both visits. The study was approved by the Institutional Review Board of the Meir Medical Center, and conducted in accordance with the Helsinki declaration for human studies. Upon recruitment, participants were examined by the attending physician at the Child Health and Sports Center and only pre pubertal (Tanner stage 1) children were included. None of the subjects had an organic disease, and none of the subjects were taking any medications that might interfere with growth or weight control or exercise tolerance (e.g. corticosteroids, thyroid hormone substitution, recombinant growth hormone etc.).

**Table 1:** Anthropometric characteristics and activity patterns of the study participants

	All *n* = 24	F *n* = 12	M *n* = 12
Age (Months)	117.4 ± 19.8	117.3 ± 15.8	117.5 ± 25.0
Weight (kg)	31.8 ± 7.0	30.6 ± 7.1	33.0 ± 7.0
Height (cm)	138.5 ± 9.4	135.4 ± 8.4	141.7 ± 9.5
BMI (kg/m^2^)	16.3 ± 1.8	16.4 ± 2.1	16.2 ± 1.5
BMI percentile (%)	40.0 ± 27.7	40.8 ± 29.5	39.2 ± 27.2
Fat percentage (%)	17.4 ± 5.0	18.4 ± 5.6	16.4 ± 4.4
Intense PA (h/week)	3.4 ± 1.8	3.2 ± 1.4	3.5 ± 2.3
Moderate PA (h/week)	2.3 ± 1.2	2.5 ± 1.1	2.2 ± 1.3
Light PA (h/week)	2.0 ± 2.7	3.0 ± 3.3	0.9 ± 1.2
Godin (Total leisure activity score)	47.9 ± 21.1	50.8 ± 20.7	45.1 ± 22.1
TV Screen time (h/day)	1.8 ± 1.0	1.6 ± 0.8	2.0 ± 1.2
Computer screen time (h/day)	1.5 ± 0.9	0.9 ± 0.6	2.1 ± 0.8
Total screen time (h/day)	3.4 ± 1.4	2.7 ± 1.0	4.1 ± 1.5

The participants were tested twice and were used as own control group, with each child being compared to him/herself.

### Design and procedure

In this study, we compared the influence of information on endurance capacity in normal weight children. Each participant performed a treadmill exercise stress test twice under identical conditions (same time of the day, room temperature, same examiner) except for the difference in the information provided regarding a drink consumed prior to testing: standard information vs. deliberate positive information. Before each testing session, the participants drank a glass of a drink. In one session, he or she were informed they were drinking water whereas, in the other session, the drink (water) was described by the researchers as a drink that increases energy levels, strengthens muscle and therefore likely to improve exercise performance. The water bottles were also styled differently for the two sessions—during the standard information session, plain transparent water bottles were used, whereas during the deliberate positive information sessions, the water bottles were opaque and blue-colored and included a label proclaiming the content to be an energy drink that strengthens muscles and improves athletic performance. The order of the provided information was randomized by a computerized random allocation generator so that half the children started with the standard information and half with the deliberate positive information. Examiners were blinded to the order of the drink consumed. Twenty minutes after consuming the drink, the participant went on the treadmill and started the exercise test.

### Measures

#### Anthropometric measurements

Standard calibrated scales (Seca 767, Hamburg, Germany) and stadiometers (Seca 240, Hamburg, Germany) were used to determine height, weight and BMI. BMI-for-age percentile was calculated according to the Center for Disease Control growth charts [8].

Fat percentage was evaluated by bioelectrical impedance analysis, using the Tanita BC-418 Segmental Body Composition Analyzer (Tanita, Illinois, USA).

#### Endurance capacity

Endurance capacity was assessed using a progressive treadmill exercise test. All subjects were familiarized with the treadmill for 5 min and performed a warm-up of 1 min at a speed of 2.2 miles per hour, with no incline. Exercise started at a speed of 2.2 miles per hour, with an incline of 10 degrees. The exercise intensity was enhanced every 2 min by increasing the elevation of the treadmill by 2.5 degrees (up to an incline of 22.5 degrees). Then the treadmill speed was increased by 0.6 miles per hour every 2 min [9]. As stated, each subject performed the test twice, using the same protocol, at the same time of the day—once believing they drank water, and once believing they drank an energy drink, at a random order. Subjects were encouraged throughout the test by the staff and exercised to the limit of their tolerance. Endurance time was measured from the end of warm up to exhaustion. Heart rate was measured using the Polar H10 heart rate monitor. Recovery time was measured as the time from the end of exercise until heart rate reached 100 bpm. The rate of perceived exertion was evaluated every minute (during test and recovery) using the Borg scale [10], average and peak RPE were calculated from the reported data.

#### Habitual activity and screen time assessment

The weekly habitual physical activity of the participants was assessed using the Godin leisure time physical activity questionnaire [11]. Each type of activity was scored according to an estimated MET score, and the final weighted score was calculated according to the formula (9 × frequency of strenuous activity) + (5 × frequency of moderate activity) + (3 × frequency of light activity). In addition, each participant was asked to list his/her weekly time spent watching television and playing computer games (screen time).

### Statistical analysis

Paired T-test was used to assess the effect of placebo administration on exercise test results. For each of the variables we performed a bivariate linear regression of the difference between the two measured groups and the potential covariate. To determine whether the result was not a result of low statistical power we also visually observed the boxplots of the distributions of the differences for different values of the covariates, to determine that the medians were not greatly different.

Data are presented as mean ± SD. Significance was set at an alpha level of *p* < 0.05.

## Results

Baseline anthropometrics and physical activity measures are presented in Table 1. As expected in pre-pubertal children, no significant gender differences were found.

**Fig. 1:**
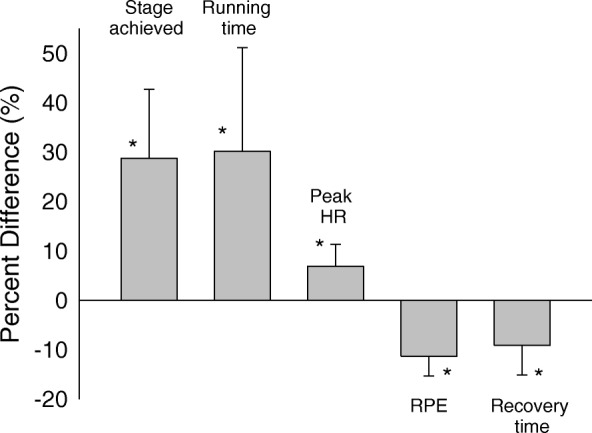
Percent difference between Placebo and water (control). A significant difference was found in stage achieved, running time and peak heart rate (HR). Drinking the placebo drink led to a lower rate of perceived exertion (RPE) and faster recovery time

The water vs placebo exercise test results are presented in Table 2 and Fig. 1. Following consuming the placebo drink, children demonstrated significantly higher peak heart rate, higher exercise test stage achieved and longer time of exercise to exhaustion. Although the exercise duration was longer, exercise test stage was higher and peak heart rate was higher, the reported average and peak rate of RPE were significantly lower following the placebo consumption, and the recovery time for the placebo group was significantly shorter.

**Table 2:** Effect of the water versus “placebo” on physical activity (PA) measures (*p < 0.05)

	Water *n* = 24	Placebo (“Energy”) *n* = 24	*P* value
Initial heart rate (bpm)	94.8 ± 7.4	95.0 ± 7.6	0.8
Ergometry phase	6.0 ± 1.3	7.6 ± 1.3*	< 0.001
Running time (sec)	700.1 ± 155.2	893.3 ± 150.1*	< 0.001
Maximal heart rate (bpm)	177.9 ± 13.6	189.8 ± 12.2*	0.003
Peak RPE	18.3 ± 1.4	16.2 ± 1.5*	< 0.001
Average RPE	12.1 ± 1.4	10.7 ± 1.5*	< 0.001
Recovery time (sec)	106.7 ± 18.6	96.7 ± 17.8*	< 0.001

## Discussion

The present study examined the effect of placebo on the results of an exercise aerobic stress test in normal weight children. We found that the use of deliberate positive information before an exercise test (in this case, water described as a drink that strengthens muscles and increases energy) led to a significant increase in running duration and the maximal heart rate achieved. In addition, we found that although performing a longer and more intense exercise test, the use of placebo was associated with a lower rate of RPE during the activity and a significantly shorter recovery time.

In the past few years, several studied tested the power of placebo effect in the context of physical activity [12], with the assumption that the placebo effect can contribute to the effect of physical activity on the body and constitute a potent factor in this context. These studies examined different types of placebo, but primarily focused on placebo in the form of information. Most studies focused on adults, few were done on adolescents [12] and to the best of our knowledge, none have been conducted on pre pubertal children [4].

In an adult study [12], female hotel room attendants were informed that their daily work constitutes healthy physical activity as recommended for their age group. A control group was not given this information. After 4 weeks, the informed group showed a decrease in body weight, blood pressure, body fat, waist-to-hip ratio, and body mass index, even though there was no change in their workload and they did not introduce any new physical activity into their daily routine. The control group showed no significant change. The authors concluded that these improved health metrics are due to the informed room attendants’ change in perception following the information they received. This study supports the assumption that the placebo effect may have an integral part in physical activity promotion and its influence on health.

In two other studies [13, 14], weightlifters consumed placebo pills they believed to be anabolic steroids and received positive information stating that the pills would improve motor performance. Later, the researchers revealed to the subjects that they merely received a placebo and not real steroids. The results found significant improvements in motor performance, such as heavier weights lifted and an increased exercise repetition rate, when the weight lifters believed they were taking steroids. Conversely, these improvements disappeared when the weight lifters discovered they had merely taken a placebo**.** Our study also found that in prepubertal children, the deliberate positive information regarding a drink consumed prior to exercise improved significantly running stage, running duration and led the children to reach a higher peak heart rate.

The mechanisms behind the placebo effect are yet to be delineated, but one of the most prominent ones is expectation; namely, the expectation of reward [15]. The term expectation of reward describes the phenomenon in which hope for improvement, and the belief in it, brings it about. The subject holds this expectation consciously based on past experience or for other reasons, such as persuasion, belief, learning or an explanation [2].

In sports, Beedie et al., [16] also focused on the expectation of a reward mechanism. In their study, cyclists performed a cycling activity after drinking a caffeine-free placebo drink. Different groups received different information, suggesting they were receiving either a caffeine-free drink, a drink with a low dose of caffeine (4.5 mg/kg), or a drink with a high dose of caffeine (9 mg/kg). Accordingly, subjects who believed they had received a high dose of caffeine showed an improvement, subjects who believed they had received a low dose of caffeine showed a smaller improvement, and subjects who believed they received a caffeine-free drink showed no significant improvement. Later, the researchers revealed to the subjects that all of them received only a caffeine-free placebo, and the subjects’ performance decreased.

Expectation of reward is known to affect pain and anxiety. The expectation of a negative outcome may cause subjects to anticipate a threat, and thus to increase anxiety, while the expectation of a positive outcome may reduce anxiety, and activate the neural networks of the brain’s associated with positive reward mechanisms [15]. This idea is clearly observed in studies of the analgesic effects of placebos, as placebo reduces anxiety, and in turn, reduced levels of anxiety leading to higher pain tolerance [17]. Studies show that subjects that expected a positive treatment effect experienced a more significant change in the brain’s metabolic activity compared to a subject who believed they were receiving a placebo, despite both groups receiving a medication with a known inherent bio-chemical effect [18]. Our study also found a decrease in the rate of perceived exertion (RPE) following the administration of the placebo. Despite the fact that subjects reached higher running stages, longer running time and a higher peak heart rate, they reported less physical exertion.

One can easily apply the same logic to the placebo effect on physical activity, the subjects’ expectations could have reduced their anxiety about the stress test, leading to a better tolerance of the exercise, and as a consequence to a faster recovery.

Children today do not meet the physical activity recommendations for their age [6, 7]. Low levels of physical activity may increase future metabolic risk in both normal weight and obese children [7]. Improving the experience of exercise, by reducing stress, and improving the duration and effectiveness of physical activity is of utmost importance. Using the placebo effect may be a promising tool.

Our results also highlight the possible bias with interpreting the results of a “maximal” exercise testing in children, since placebo information as well as other motivating aids and fatigue distractors may lead the child to a better performance [19].

Limitations to our study included the relatively small sample size of pre-pubertal normal weight children, and the fact that the study was performed in a laboratory setting. Moreover, additional components of fitness were not evaluated in our study.

## Conclusions

Our study clearly showed that children were highly affected by the placebo effect, and were able to better perform in an exercise test, and recover faster. These effects were achieved for the first time in a laboratory setting. Further studies are needed to explore if using placebo in a “real life” setting, or in pediatric populations in need (e.g. overweight and obese children), will lead to similar beneficial effects. Moreover, the ability to maintain the placebo effect in children should also be evaluated.

## Abbreviations

BMI: Body mass index
BPM: Beats per minute
PA: Physical activity
RPE: Rate of perceived exertion

## References

[1] Price DD, Finniss DG, Benedetti F (2008). A comprehensive review of the placebo effect: recent advances and current thought. Annu Rev Psychol.

[2] Koshi EB, Short CA (2007). Placebo theory and its implications for research and clinical practice: a review of the recent literature. Pain Pract.

[3] Bootzin RR, Bailey ET (2005). Understanding placebo, nocebo, and iatrogenic treatment effects. J Clin Psychol.

[4] Weimer K, Gulewitsch MD, Schlarb AA, Schwille-Kiuntke J, Klosterhalfen S, Enck P (2013). Placebo effects in children: a review. Pediatr Res.

[5] Eliakim A, Nemet D (2010). Exercise training, physical fitness and the growth hormone-insulin-like growth factor-1 axis and cytokine balance. Med Sport Sci.

[6] Nemet D, Cooper DM (2002). Exercise, diet, and childhood obesity: the GH-IGF-I connection. J Pediatr Endocrinol Metab.

[7] Ekelund U, Luan J, Sherar LB, Esliger DW, Griew P, Cooper A (2012). International Children's Accelerometry database C: moderate to vigorous physical activity and sedentary time and cardiometabolic risk factors in children and adolescents. JAMA.

[8] Kuczmarski RJ, Ogden CL, Grummer-Strawn LM, Flegal KM, Guo SS, Wei R, Mei Z, Curtin LR, Roche AF, Johnson CL. CDC growth charts: United States. Adv Data. 2000:1–27. 11183293

[9] Nemet D, Oren S, Pantanowitz M, Eliakim A (2013). Effects of a multidisciplinary childhood obesity treatment intervention on adipocytokines, inflammatory and growth mediators. Horm Res Paediatr.

[10] Borg GA (1982). Psychophysical bases of perceived exertion. Med Sci Sports Exerc.

[11] Godin G, Shephard RJ (1985). A simple method to assess exercise behavior in the community. Can J Appl Sport Sci.

[12] Crum AJ, Langer EJ (2007). Mind-set matters: exercise and the placebo effect. Psychol Sci.

[13] Kalasountas V, Reed J, Fitzpatrick J (2007). The effect of placebo-induced changes in expectancies on maximal force production in college students. J Appl Sport Psychol.

[14] Maganaris CN, Collins D, Sharp M (1999). Expectancy Effects and Strength Training: Do Steroids Make a Difference?.

[15] Benedetti F, Carlino E, Pollo A (2011). How placebos change the patient's brain. Neuropsychopharmacology.

[16] Beedie CJ, Stuart EM, Coleman DA, Foad AJ (2006). Placebo effects of caffeine on cycling performance. Med Sci Sports Exerc.

[17] Benedetti F, Amanzio M, Casadio C, Cavallo A, Cianci R, Giobbe R, Mancuso M, Ruffini E, Maggi G (1997). Control of postoperative pain by transcutaneous electrical nerve stimulation after thoracic operations. Ann Thorac Surg.

[18] Volkow ND, Wang GJ, Ma Y, Fowler JS, Zhu W, Maynard L, Telang F, Vaska P, Ding YS, Wong C, Swanson JM (2003). Expectation enhances the regional brain metabolic and the reinforcing effects of stimulants in cocaine abusers. J Neurosci.

[19] Tenenbaum G, Lidor R, Lavyan N, Morrow K, Tonnel S, Gershgoren A, Meis J, Johnson M (2004). The effect of music type on running perseverance and coping with effort sensations. Psychol Sport Exerc.

